# Canonical and early lineage-specific stem cell types identified in planarian SirNeoblasts

**DOI:** 10.1186/s13619-021-00076-6

**Published:** 2021-03-19

**Authors:** Kaimeng Niu, Hao Xu, Yuanyi Zhou Xiong, Yun Zhao, Chong Gao, Chris W. Seidel, Xue Pan, Yuqing Ying, Kai Lei

**Affiliations:** 1 Westlake Laboratory of Life Sciences and Biomedicine, Key Laboratory of Growth Regulation and Translational Research of Zhejiang Province, School of Life Sciences, Westlake University, Hangzhou, Zhejiang China; 2 Institute of Biology, Westlake Institute for Advanced Study, Hangzhou, Zhejiang China; 3 College of Life Sciences, Zhejiang University, Hangzhou, Zhejiang China; 4 Stowers Institute for Medical Research, Kansas City, MO 64110 USA

**Keywords:** Planaria, Regeneration, Neoblast, SiR-DNA, scRNA-seq

## Abstract

**Background:**

The pluripotent stem cells in planarians, a model for tissue and cellular regeneration, remain further identification. We recently developed a method to enrich *piwi-1*+ cells in *Schmidtea mediterranea*, by staining cells with SiR-DNA and Cell Tracker Green, named SirNeoblasts that permits their propagation and subsequent functional study in vivo. Since traditional enrichment for planarian neoblasts by Hoechst 33342 staining generates X1 cells, blocking the cell cycle and inducing cytotoxicity, this method by SiR-DNA and Cell Tracker Green represents a complementary technological advance for functional investigation of cell fate and regeneration. However, the similarities in heterogeneity of cell subtypes between SirNeoblasts and X1 remain unknown.

**Results:**

In this work, we performed single cell RNA sequencing of SirNeoblasts for comparison with differential expression patterns in a publicly available X1 single cell RNA sequencing data. We found first that all of the lineage-specific progenitor cells in X1 were present in comparable proportions in SirNeoblasts. In addition, SirNeoblasts contain an early muscle progenitor that is unreported in X1. Analysis of new markers for putative pluripotent stem cells identified here, with subsequent sub-clustering analysis, revealed earlier lineages of epidermal, muscular, intestinal, and pharyngeal progenitors than have been observed in X1. Using the *gcm* as a marker, we also identified a cell subpopulation resided in previously identified *tgs-1*+ neoblasts. Knockdown of *gcm* impaired the neoblast repopulation, suggesting a function of *gcm* in neoblasts.

**Conclusions:**

In summary, the use of SirNeoblasts will enable broad experimental advances in regeneration and cell fate specification, given the possibility for propagation and transplantation of recombinant and mutagenized pluripotent stem cells that are not previously afforded to this rapid and versatile model system.

**Supplementary Information:**

The online version contains supplementary material available at 10.1186/s13619-021-00076-6.

## Background

The capacity for regeneration is widely distributed throughout the animal kingdom. Identification of the cell lineage types and their composition among larger populations of regenerative cells have become essential steps in the process of dissecting the molecular mechanisms controlling tissue regeneration (Gerber et al. 2018; Hou et al. 2020; Reddien 2018).

The planarian *Schmidtea mediterranea* has been widely studied as an animal model for tissue regeneration due to its capability of rapid whole-body regeneration (Elliott and Sánchez Alvarado 2013; Reddien 2018). The adult stem cell neoblasts consist of the cellular origin for all cell types in homeostasis and regeneration. Identification of lineage specific cell types within the neoblasts is necessary to understand the cellular basis of planarian regeneration. Therefore, the isolation and application of these cells for downstream studies such as cell culture and genome editing have become essential for further research on cell lineage tracing and cell type-specific gene function. However, due to the cytotoxicity of Hoechst 33342 used in the traditional isolation method, alternative methods are needed to enrich neoblasts for propagation (Lei et al. 2019; Wagner et al. 2011). In our previous efforts to culture neoblasts, we combined the DNA staining dye SiR-DNA and Cell Tracker Green in order to enrich *piwi-1*+ neoblasts, thus designated SirNeoblasts (Lei et al. 2019). The primary advantage of this strategy over Hoechst 33342 is the lower DNA binding affinity of SiR-DNA, which retains cell viability, allowing subsequent downstream functional assays such as cell transplantation (Bucevicius et al. 2018). Using this strategy, neoblasts can be used for cell culture with continued pluripotency in further examinations. However, we have not yet verified whether SirNeoblasts consist of the similar cell types as X1, which is necessary for comparability between previous work in X1 cells and SirNeoblasts.

Single cell RNA sequencing (scRNA-seq) and related analytical methods have become essential tools for understanding the cellular dynamics of organismal development, disease progression, and tissue regeneration (Birnbaum 2018). Moreover, these molecular techniques provide a sophisticated and data-rich means of identifying novel cell types and projecting the cell lineage trajectory in a systematic manner. Planarian neoblasts were first identified by the pan-neoblast marker *piwi-1*+ (Reddien et al. 2005). To date, seven scRNA-seq studies in planarians have been reported (Fincher et al. 2018; Molinaro and Pearson 2016; Plass et al. 2018; Swapna et al. 2018; van Wolfswinkel et al. 2014; Wurtzel et al. 2015; Zeng et al. 2018). Using the first generation Fluidigm platform, three major subtypes of neoblasts, σ, δ and γ, were distinguished (van Wolfswinkel et al. 2014), followed by later identification of the neuronal progenitor *nu* neoblasts (Molinaro and Pearson 2016). More recently, clusters of progenitor lineages have been recognized in X1 (Zeng et al. 2018). Nb2 cells expressing *tgs-1* were proposed as the prospective pluripotent stem cells. Although SirNeoblasts are enriched with *piwi-1*+ cells at a similar level as X1, the cellular differences between X1 and SirNeoblasts have not yet been carefully scrutinized (Lei et al. 2019). We hypothesized that SirNeoblasts contained comparable lineage-specific heterogeneity to X1 cells, which would ultimately permit their use as a reliable, comparable resource for functional and regulatory investigation of cell regeneration.

To compare the similarities and differences between SirNeoblasts and X1, we conducted scRNA-seq to analyze the cell types within SirNeoblast populations through the identification of differential gene expression patterns specific to progenitor lineages. To this end, we combined our RNAseq data from SirNeoblasts with publicly available relative expression data of X1, and subsequently confirmed that all of the previously identified lineage-specific progenitor clusters found in X1 were also present in SirNeoblasts, as well as an early muscle progenitor not yet observed in X1 populations. Furthermore, subclustering of two clusters within SirNeoblasts also identified four types of early lineage specific progenitors and several lineage-specific marker genes that have not yet been reported. We foresee the wide adoption of these SirNeoblast cells for genetic analyses of several fundamental regulatory and functional questions regarding regeneration and cell fate determination in the planarian model.

## Methods

### Planarian maintenance

Asexual *Schmidtea mediterranea* (strain CIW4) specimens were maintained and propagated at 20 °C in 1X Montjuïc salts, as previously described (Newmark and Sánchez Alvarado 2000). All animals were randomly selected at 8 ~ 10 mm for flow cytometry and 2 ~ 3 mm for fluorescence in situ hybridization and RNAi, then starved for 7–10 days prior to the experiments. Animals were exposed to 12.5 Gy for sublethal irradiation experiments using a RS2000 pro X-ray irradiation apparatus.

### Flow cytometry of SirNeoblasts

In order to obtain isolated SirNeoblasts, the tails of the planarians (> 8 mm in length) were amputated, then pooled and rinsed in calcium and magnesium free buffer with 1% bovine serum albumin (CMFB). Cells were macerated by rocking in the tube on a rotating platform for 20 min with agitation every 3 min. After filtering the macerated cells through a 70 μm cell-strainer cap, the dispersed cells were centrifuged at 290 x g for 10 min. Cells were then resuspended in isotonic planarian medium (IPM) with 10% Fetal Bovine Serum (FBS, CellMax SA211.02) for SiR-DNA staining by incubation in SiR-DNA (1 μM, Cytoskeleton Inc., CY SC007) for 1 h and Cell Tracker green CMFDA stains (2.5 μg/ml, Thermo Fisher Technologies, C7025) for 10 min. Target cells were sorted using a BD Influx cell sorter equipped with a 100 tip and purity sort mode.

### Single cell sequencing and analysis

The cells captured by flow cytometry were sequenced to 322 million reads, and the reads were aligned to the planarian transcriptome by cellranger v 2.1.0 (Robb et al. 2015). Seurat v3 (Butler et al. 2018) was used to cluster cells with the parameter (PCS = 10, resolution = 0.6) after data cleaning in R 3.6.3. The R code is available upon request. The t-distributed stochastic neighbor embedding (t-SNE) was then used to visualize clustering distance. Markers were calculated with the FindAllMarkers function and were subsequently used for cluster annotation. The pseudotime analysis of SirNeoblasts is done by diffusion pseudotime (DPT) (Haghverdi et al., 2016).

The X1 scRNA-seq data (GSE107873) was download from GEO. This dataset was then used to integrate with cleaned SirNeoblast scRNA-seq data following a standard integration workflow in Seurat v3. The functions FindIntegrationAnchors (dims = 10) and IntegrateData (dims = 10) were used to find anchors and integrate two samples. Seventeen clusters were clustered and annotated.

Cells from C3 and C5 clusters were used for sub-clustering (PCS = 10, resolution = 0.6) and nine sub-clusters were resolved. Slingshot was used to conduct pseudotime analyses with the marker genes for lineage construction (Street et al. 2018). These marker genes from lineage specific clusters were also used as input for Mfuzz analysis (Kumar and Futschik 2007). The pathway enrichment analysis was done by R package clusterProfiler (Yu et al. 2012).

### T4P cloning and probe synthesis

All of the cloned transcripts were referenced from Planosphere (Davies et al. 2017). The cDNA library of asexual CIW4 was used as template for cloning genes. The PCR products of each transcript were ligated into the T4p vector (Adler et al. 2014). The RNA probes were synthesized using a reverse transcription reaction with T7 RNA polymerase (Promage PAP 2077), transcription buffer, 10 × RNA labeling (DIG RNA Labeling Mix: Roche 11,277,073,910; Fluorescein RNA Labeling Mix: Roche 11,685,619,910; DNP-11-UTP: PerkinElmer NEL555001EA), and template plasmid DNA.

### Phylogenic analysis

Sequences of *gcm* (*SMED30023953*) homologous genes in several species were retrieved from Uniport (https://www.uniprot.org/) for evolutionary analysis, including Q9NP62, P70348, N1PB97, Q27403, K7J7U6, A0A2C9JHH9, and V4A9N6. ClustalW was used to perform multiple sequence alignment of protein sequences in MEGAX v10.1.1. Sequence trimming was performed with crafts. Phylogenetic trees were constructed by Neighbor-joining method with a bootstrap value 1000 and the poisson model.

### Whole mount in situ hybridization and antibody staining

Whole mount fluorescent in situ hybridization (FISH) was performed as previously described (King and Newmark 2013; Pearson et al. 2009). The probe for *piwi-1* was used at 1:1000 dilution ratio. Other probes (*tgs-1*, *zfp-1*, *soxP-3*, *gata4/5/6*, *hoxb7*, *SMED30028798*, *pou2/3*, *gcm*, *ski-3*) were used at 1:500 dilution ratio. Anti-phospho-Histone H3 (Ser10) (H3P) antibody (Abcam, ab32107) was used at 1:1000 dilution ratio.

### Microscopy and image analysis

Living worms and FISH samples were imaged with a Leica M205 FA stereomicroscope. Immunofluorescence and FISH samples were imaged with a Nikon A1 R HD25 or Nikon Csu-W1. Images were processed with Fiji (version 2.0.0) and Adobe Photoshop (cc 2018).

## Results

### SirNeoblasts are heterogenous and consist of known lineages

**Fig. 1:**
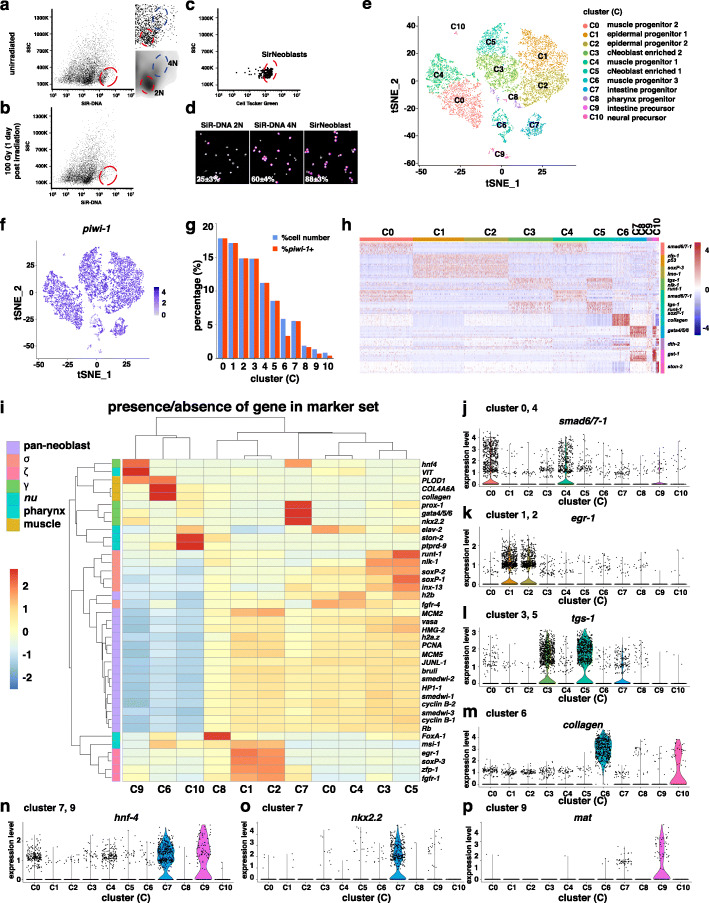
SirNeoblasts contain the major cell types found in X1 neoblasts. **a**-**b** Flow cytometry plots of cells from unirradiated planarians (**a**) and irradiated planarians (**b**), stained with SiR-DNA. Red circle indicates the irradiation sensitive cells. Blue circle indicates cells (4 N) for further enrichment. **c** Flow cytometry plot showing the gating of SirNeoblasts (cells in red circle) by combining the SiR-DNA and Cell Tracker Green. **d** Images and percentages of *piwi-1+* cells in sorted SiR-DNA stained 2 N, 4 N, and SirNeoblsts. **e** Single cell clustering for SirNeoblasts. **f** tSNE plot showing cells with high *piwi-1*+ expression (blue). **g** Proportions of total cells and high *piwi-1+* expression cells in each cluster. **h** Heatmap of markers for each cluster. **i** Heatmap correlation analysis for traditional neoblast markers. **j**-**p** Violin plots showing the enrichment and expression levels of each neoblast marker

To identify differences between SirNeoblasts and X1 cells, we first tested whether the SirNeoblasts are irradiation sensitive. We found that both 2 N and 4 N SiR-DNA+ cells were similarly sensitive to irradiation (Fig. 1a and b, Supplemental Fig. 1A). Consistent with our previous report, 2 N and 4 N SiR-DNA+ populations contained 25 ± 3% and 60 ± 4% *piwi-1*+ cells, while SirNeoblasts contained 88 ± 3% *piwi-1*+ cells (Fig. 1c and d) (Lei et al. 2019). These results suggest that SirNeoblasts exhibit comparable irradiation sensitivity to that of X1 cells.

We then performed scRNA-seq to identify cell types in SirNeoblasts. Using Seurat analysis, 11 clusters were identified (Fig. 1e). Consistent with our initial findings, 96% of the cells were *piwi-1*+ cells (Fig. 1f and g). The top 10 marker genes for each cluster were then used to distinguish each cluster (Fig. 1h and Additional file 5: Table S1). Classic neoblast subtype markers such as σ, δ, γ, *nu*, muscular, and pharyngeal progenitors were then used to probe the identity of each cluster (Fig. 1i-p)(Molinaro and Pearson 2016; van Wolfswinkel et al. 2014; Zeng et al. 2018). These analyses revealed that the C3 and C5 cluster populations were most likely to carry the *tgs-1*+ pluripotent stem cells. The C0 and C4 clusters were identified as early muscular progenitors, while C6 consisted of late muscular progenitors. The C1 and C2 clusters were characterized as epidermal progenitors, C8 cluster as parenchymal and pharyngeal progenitors, C7 cluster as the intestinal progenitors, and C9 cluster was found to contain the intestinal precursors. Lastly, the C10 cluster was determined to contain neural precursors. The expression levels of pan-neoblast markers such as *piwi-1*, *PCNA*, *h2b*, and *cyclin B2* in each cluster supported the differences between pluripotent stem cells, early progenitors, late progenitors, and lineage-specific precursors (Supplemental Fig. 1B). Pseudotime prediction results also suggested that C5 cluster consists of more pluripotent stem cells (cNeoblasts) than C3 cluster (Supplemental Fig. 1C). Although the difference between C0 and C4, C1 and C2, or C3 and C5 is subtle, pseudotime trajectory and mRNA levels of other marker genes such as collegan suggest potentially different status of these cells (Fig. 1h and Supplemental Fig. 1C). We therefore used numeric letter to indicate their difference.

To further examine the consistency with previously reported Nb subtypes in X1, differential expression of X1 markers was examined in scRNA-seq data to determine if these markers were also able to distinguish SirNeoblast clusters (Supplemental Fig. 1D) (Zeng et al. 2018). Results of violin plot analysis showed that *soxP-3*, *egrG*, *runt-1*, *Imo-1*, *myosin*, *gata4/5/6*, and *ston-2* all served as reliable markers in SirNeoblasts (Supplemental Figs. 1E-K), suggesting that SirNeoblasts contain similar cell types to X1 cells.

### SirNeoblasts enrich an additional cluster of early muscular progenitors compared to X1

**Fig. 2:**
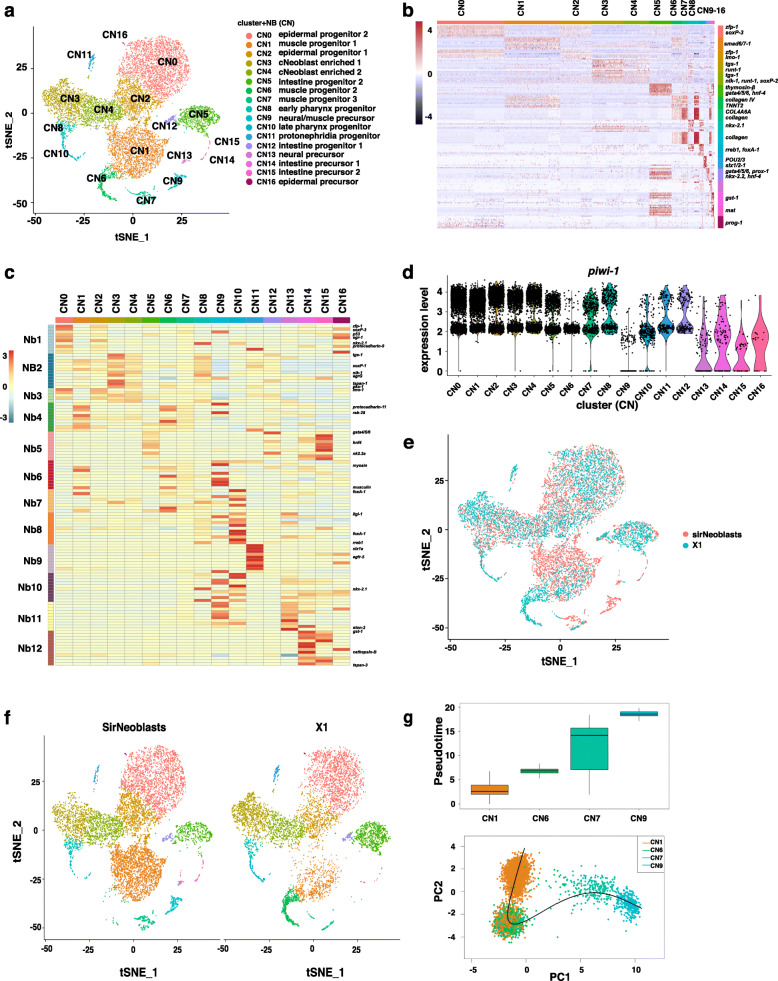
Integration of scRNA-seq data from SirNeoblasts and X1. **a** tSNE plot showing clusters after integration of two datasets. **b** Heatmap of markers for each cluster. **c** Heatmap correlation analysis for markers in X1 scRNA-seq data. **d** Violin plot showing the *piwi-1*+ distribution in each cluster. **e** tSNE comparison of entropy for each cluster in integrated datasets. **f** tSNE plot of the proportions of total cells distributed in each cluster for SirNeoblasts and X1. **g** Pseudotime trajectory analysis of muscle lineages

Although the cluster markers in X1 can be mapped to each cluster in SirNeoblasts, the cell types within SirNeoblasts and X1 may differ. To address this question, we integrated SirNeoblast and X1 scRNA-seq data, which led to the classification of 17 clusters across the combined two datasets (Fig. 2a). Cluster markers successfully distinguished each cluster (Fig. 2b and Additional file 6: Table S2). Classic neoblast subtype markers such as σ, δ, γ, *nu*, muscular, and pharyngeal progenitors, as well as X1 Nb markers were then used to identify the cell types of each cluster. All known lineages were confirmed to be included (Fig. 2c and Supplemental Fig. 2A). Expression levels of *piwi-1* and other pan-neoblast markers were then used to distinguish the early and late progenitors (Fig. 2d and Supplemental Fig. 2A). These results suggested that the two datasets were successfully integrated.

To observe the distribution of cells from SirNeoblasts or X1, split tSNE plots were used to visualize the populations, and the ratio of cell numbers from each cluster were compared (Fig. 2e, f and Supplemental Fig. 2B). We found that the cell types were overall very similar between SirNeoblasts and X1. X1 populations had a slightly higher proportion of *tgs-1*+ cells and intestinal progenitors than SirNeoblasts, while SirNeoblasts had a higher proportion of muscular progenitors than X1. The epidermal progenitors comprised the same proportion in both SirNeoblasts and X1. These results support that SirNeoblasts are enriched with all, or almost all, of the cell types found in the X1 population.

Close examination of the integrated data showed that the number of SirNeoblasts within the CN1, CN6, CN7, and CN9 clusters were obviously different than the number of X1 cells in these clusters. Notably, these clusters all exhibited highly differential expression of marker genes for early and late muscular progenitors (Fig. 2b and c). PCA and pseudotime analyses suggested a sequential differentiation in muscular lineage following the order of CN1-CN6-CN7-CN9 (Fig. 2g). These integrated datasets thus revealed a more complete lineage trajectory for planarian muscle cell development than previously recognized in X1.

These above findings led us to propose that isolated SirNeoblasts can thus be used for functional assays, either in vivo or for engineering proliferation, which are not possible with X1 cells.

### SirNeoblast subclusters reveal earlier lineage specific population of stem cells

**Fig. 3:**
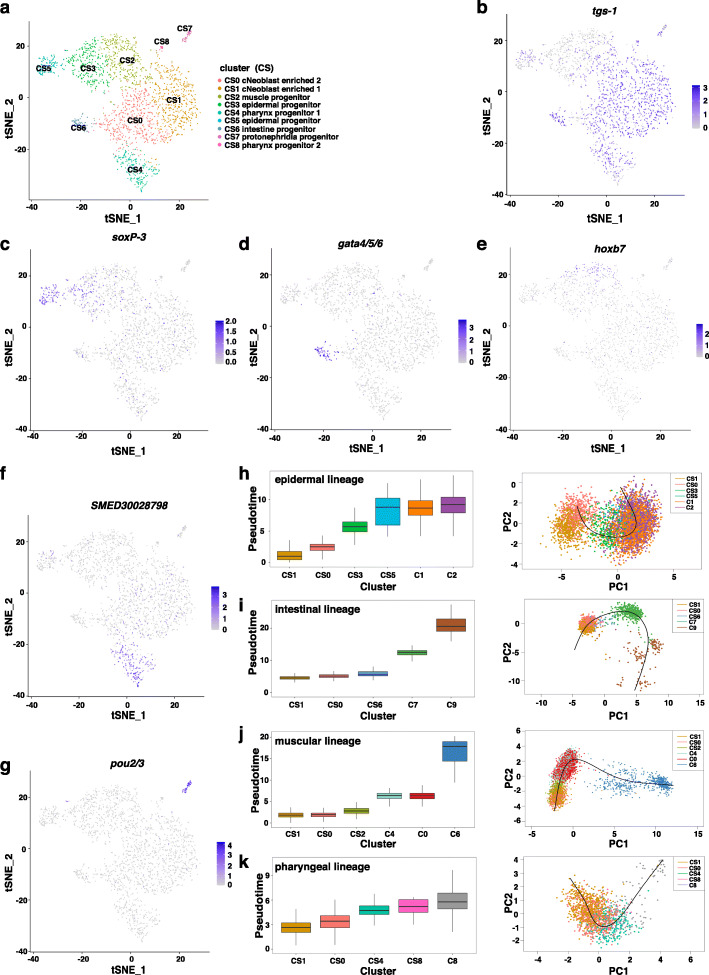
Perspective subclusters within the pluripotent stem cell populations. **a** tSNE plot of subclustering within C3 and C5 cells in SirNeoblasts. **b** tSNE plot showing *tgs-1*+ cells in blue. **c** tSNE plot showing *soxP-3*+ cells in blue. **d** tSNE plot showing *gata4/5/6*+ cells in blue. **e** tSNE plot showing *hoxb7*+ cells in blue. **f** tSNE plot showing *SMED30028798*+ cells in blue. **g** tSNE plot showing *pou2/3*+ cells in blue. **h** Pseudotime trajectory of epidermal progenitor lineage including CS1, CS0, CS3, CS5, C1, and C2. **i** Pseudotime trajectory of intestinal progenitor lineage including CS1, CS0, CS6, C7, and C9. **j** Pseudotime trajectory of muscular progenitor lineage including CS1, CS0, CS2, C4, C0, and C6. **k** Pseudotime trajectory of pharyngeal progenitor lineage including CS1, CS0, CS4, CS8, and C8

The population containing pluripotent neoblasts in X1 have been previously described as Nb2 (Zeng et al. 2018). Comparison of marker gene expression between SirNeoblasts and X1 revealed that C3 and C5 clusters in SirNeoblasts were a similar population to Nb2 cells in X1 (Fig. 1e and Supplemental Fig. 1D). However, we noticed that some epidermal progenitor and protonephridia progenitor markers were also expressed in the the C3 and C5 clusters (Supplemental Fig. 1D), leading us to hypothesize that the C3 and C5 clusters potentially contain some early stage progenitors. To test this possibility, we performed further subclustering analysis for C3 and C5 clusters, which subsequently resolved 9 subclusters (CS0-CS8) (Fig. 3a). These subclusters contained putative pluripotent stem cells (*tgs-1*+, CS0 and CS1), as well as lineage specific progenitors, including epidermal (*soxP-3*+), intestinal (*gata4/5/6*+), muscular (*hoxb7*), pharyngeal (*SMED30028798*), and protonephridia (*pou2/3*) (Fig. 3b-g). Pseudotime analyses of each lineage and dual FISH of each lineage marker gene with *tgs-1* in SirNeoblasts and planarians supported our identification of previously unrecognized tissue-specific progenitor subclusters within C3 and C5, therefore suggesting that the cell fate determination likely occurs at an earlier developmental stage than that indicated by prior studies (Fig. 3h-k, Supplemental Figs. 3A-C).

To clarify the dynamic patterns of gene expression specific to cell types, we conducted Mfuzz analysis to cluster the genes according to their changes in expression specific to each lineage (Supplemental Figs. 3D-G and Additional file 7: Table S3). The results of this analysis showed that, in contrast with markers for other clusters, several genes were specifically upregulated in the four lineages of the C3/C5 subclusters. These results suggested that several new candidate markers could be developed for the functional study of the regulation of the cell fate determination.

### SirNeoblast subclusters reveal a perspective population of pluripotent stem cells

**Fig. 4:**
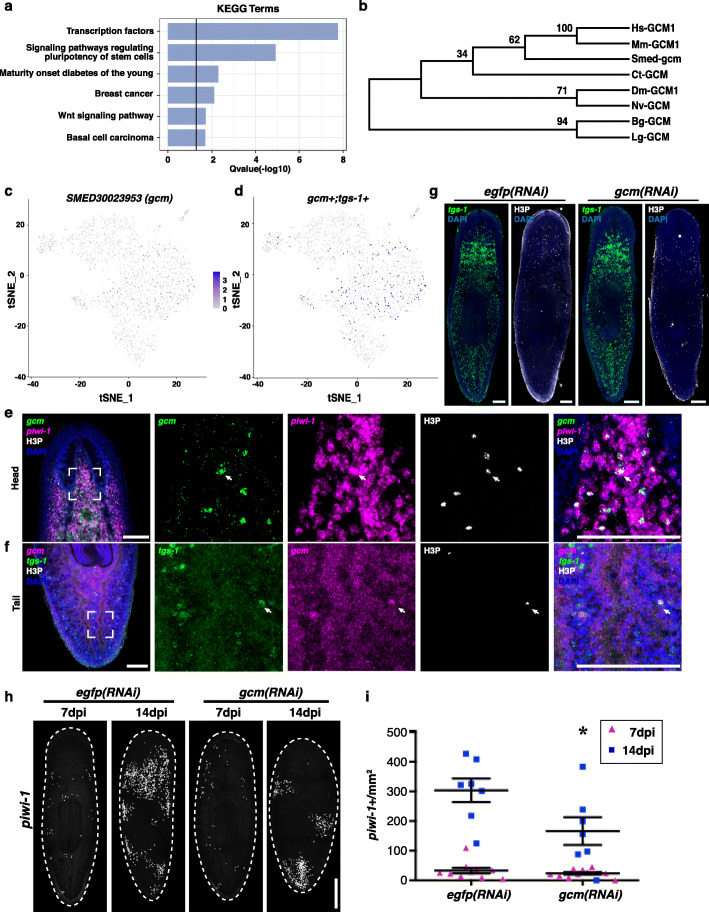
Pluripotent stem cells are enriched in CS0 and CS1 subclusters. **a** KEGG enrichment for genes in CS1 and CS0. **b** Phylogenic tree of *gcm* in the GCM transcription factor family. Dm, *Drosophila melanogaster*. Nv, *Nasonia vitripennis*. Bg, *Biomphalaria glabrata*. Lg, *Lottia gigantea*. Ct, *Capitella teleta*. Hs, *Homo sapiens*. Mm, *Mus musculus*. **c** tSNE plot showing *SMED30023953* (*gcm*) + cells in blue. **d** tSNE plot showing *gcm* + *tgs-1+* cells in blue. **e** FISH showing co-localization of *gcm* with *piwi-1*. White box indicates inset with high magnification in column second from left. *piwi-1*+ (magenta); *gcm* (green); phosphorylated histone 3 (H3P) (white); nuclei (blue) indicate antibodies/channels. Arrows indicate a *piwi-1 + gcm +* H3P+ cell. Scale bars indicate 200 μm. **f** FISH showing co-localization of *gcm* with *tgs-1*. White box indicates inset with high magnification in column second from left. *gcm* + (magenta); *tgs-1* (green); H3P (white); nuclei (blue) indicate antibodies/channels. Arrows indicate a *tgs-1 + gcm +* H3P+ cell. Scale bars indicate 200 μm. **g** Unirradiated *egfp (RNAi)* and *gcm (RNAi)* planarians stained for *tgs-1* FISH (green), anti-H3P antibody (white) and DAPI (blue). **h** *egfp (RNAi)* and *gcm (RNAi)* planarians at 7 dpi and 14 dpi stained for *piwi-1* FISH. **i** Quantification of *piwi-1+* cells/mm^2^ shows impaired recovery of neoblasts in *gcm (RNAi)* versus *egfp (RNAi)* planarians. *, *p* < 0.05

Based on these marker genes and cell lineage analyses, we then combined the CS0 and CS1 subclusters to identify markers specific to these cells. KEGG enrichment analysis suggested that the most reliable candidate markers were mainly transcription factors or were associated with signaling pathways for the regulation of pluripotency, which further supported our hypothesis (Fig. 4a and Additional file 8: Table S4). Within these marker genes, the *ski-3* transcription factor was enriched in CS0 and CS1 subclusters (Supplemental Figs. 4A-C). Fluorescence in situ hybridization revealed that *ski-3* was expressed in subsets of both neoblasts and mature nervous system cells, consistent with previous reports (Supplemental Fig. 4E) (Molinaro and Pearson 2016; Wurtzel et al. 2015). Furthermore, *ski-3* + *tgs-1*+ cells enriched in CS0 and CS1 could be detected in planarians by dual FISH (Supplemental Figs. 4D and F), suggesting a potential pluripotency of these *ski-3* + *tgs-1*+ cells. Due to a combinatory function of *ski-3* in neural lineage, the function of *ski-3* in pluripotency was hampered to be studied. On the other hand, another transcription factor *Smed-gcm* (*gcm*) belonging to the GCM family was identified (Fig. 4b), which was specifically expressed in a subset of neoblasts (Fig. 4c-e). These double positive cells were also highly associated with the cell proliferation marker labeled with anti-PH3 antibody for the G2/M cell cycle phase (Fig. 4e). Even though a small number of epithelial progenitors also expressed *gcm*, the majority of *tgs-1* + *gcm* + cells were enriched in CS0 and CS1 clusters (Fig. 4d). In addition, the *tgs-1* + *gcm* + cells in planarians were verified by dual FISH (Fig. 4f). To further examine the function of *gcm* in vivo, the numbers/densities of neoblasts were compared in *gcm (RNAi)* and *egfp (RNAi)* planarians. Even though no obvious defect was observed in homeostasis, neoblasts in *gcm (RNAi)* planarians showed impaired repopulation after sublethal irradiation compared to those in *egfp (RNAi)* controls (Fig. 4g-i). These results suggest a function of *gcm* in pluripotent stem cells in planarians.

## Discussion

### SirNeoblasts contain the similar neoblast population of X1

Historically, studies on the planarian neoblasts have relied heavily on RNA interference and Hoechst 33342 staining-based flow cytometry. However, due to the cytotoxicity caused by cell cycle blockage, Hoechst stained neoblasts cannot proliferate, which therefore limits its use in the development of cell culture research methods (Wagner et al. 2011). Previous study has used SiR-DNA combined with Cell Tracker Green to enrich *piwi-1*+ cells from planarians (Lei et al. 2019). These SirNeoblasts maintain their proliferative capacity and can rescue stem cell depleted planarians, thus providing a viable alternative to X1 cells for the study of stem cell proliferation and regeneration in the planarian model. However, the similarities between SirNeoblasts and X1 cells were not fully characterized, which is essential for their comparability between studies.

More specifically, this study endeavored to determine the full suite of cell types that comprise the SirNeoblast population through comparison of the scRNA-seq data from both SirNeoblasts and X1 cells. We found that ~ 96% of SirNeoblasts transcriptionally express *piwi-1* and contain clearly distinct populations of pluripotent stem cells and lineage-specific progenitors for epidermis, muscle, intestine, neuron, and pharynx cells. SirNeoblasts ostensibly contain all of the same cell types that have been identified in X1 cells, and in comparable proportions of each cluster. Combined with our previous report on SirNeoblast capacity for proliferation in cell transplantation, our results support the use of these cells as a reliable and comparable alternative to X1-like neoblasts for functional studies of regulation of regeneration and cell fate determination.

### Subclustering revealed early cell lineage differentiation within previously identified pluripotent stem cell types

The pluripotent stem cells are the cellular basis for planarian regeneration and the identification of these cells has been slowly determined over generations of research beginning with seminal, morphology-based characterization of neoblasts as the adult stem cells in planarians (Wolff and Dubois 1948). Subsequently, molecular markers for neoblasts were identified such as *PCNA*, *vasa*, *h2b*, and *piwi-1*, with the latter of these emerging as the most reliable (Orii et al. 2005; Reddien et al. 2005; Shibata et al. 1999). With the introduction of increasing numbers of molecular markers, the heterogeneity of neoblasts became widely apparent (Rink 2013; Tanaka and Reddien 2011). Single cell transplantation experiments showed the existence of pluripotent stem cells within neoblasts (Wagner et al. 2011), leading thereafter to the identification of neoblast subtypes σ, γ, δ, and *nu* through single cell analysis (Molinaro and Pearson 2016; van Wolfswinkel et al. 2014). More recently, the 10x Genomics platform enabled the resolution of 12 clusters of neoblasts and Nb2 was proposed as the population enriching the pluripotent stem cells (Zeng et al. 2018). In our current study, we identified similar cell types within SirNeoblast populations. Using molecular markers for Nb2 from X1, a population of the C3 and C5 clusters were identified in SirNeoblasts. However, we also found that this population could be further subclustered into several lineage-specific categories, thus indicating that cell fate determination occurred earlier than previously thought in these pluripotent stem cells. Because SirNeoblasts and X1 were isolated by different staining strategies, we could not know whether the Nb2 population from X1 contained similar lineage-specific clusters. Moreover, in contrast to X1, individual cells from this newly identified pluripotent stem cell population could be isolated for further functional studies.

### scRNA-seq of SirNeoblasts facilitates the dissection of the regulatory mechanisms of planarian stem cells

The knowledge of neoblast maintenance and differentiation is originated primarily from studies of pan-neoblast specific regulators and lineage specific transcription factors (Reddien 2013, 2018; Tu et al. 2015; Wagner et al. 2012; Wurtzel et al. 2015; Zhu et al. 2015). Investigation of the regulatory mechanisms that control the early stages of cell fate specification were in the nascent stages at the time the cellular diversity within neoblasts was first recognized (Molinaro and Pearson 2016; van Wolfswinkel et al. 2014; Zeng et al. 2018). In our current study, subclustering based on differential gene expression not only revealed a putative population of pluripotent stem cells and early lineage specific clusters, but also identified genes enriched in each cluster. Notably, we found several genes that are transiently upregulated in specific cell lineages, suggesting that they may contribute necessary functions to the induction of the cell fate specification. Detailed study of these genes may disclose novel mechanisms for neoblast maintenance and differentiation in planarians.

## Supplementary Information

**Additional file 1: Supplemental Fig. 1.** (A) Flow cytometry plots showing the gating steps to enrich SirNeoblasts. (B) Violin plots showing the enrichment and expression levels of pan-neoblast markers. (C) Pseudotime snapshot of lineage analysis for perspective pluripotent stem cells with epidermal (purple), intestinal (blue), muscular (orange) progenitors and C5 (red), C3 (green) populations. (D) Heatmap correlation analysis for markers from X1 scRNA-seq data. (E-K) Violin plots showing the enrichment and expression levels of each neoblast marker.

**Additional file 2: Supplemental Fig. 2.** Verification of markers from X1 single cell data. (A) Heatmap correlation analysis for traditional markers. (B) Proportion of total cells and *piwi-1*+ cells distributed in each cluster.

**Additional file 3: Supplemental Fig. 3.** Gene expression dynamics in each progenitor lineage. (A) FISH of *tgs-1* (white) with lineage marker genes (magenta) including *zfp-1*, *soxP-3*, *gata4/5/6*, *hoxb7*, *SMED30028798* (28798), and *pou2/3* in SirNeoblasts, respectively. The numbers indicate the number of positive cells versus the number of total cells counted. Scale bars indicate 10 μm. (B) FISH of *gata4/5/6* (magenta) with *tgs-1* (green) in planarians. Scale bars indicate 50 μm. Arrows indicate the *tgs-1 + gata4/5/6+* cells. (C) FISH of *pou2/3* (magenta) with *tgs-1* (green) in planarians. Scale bars indicate 50 μm. Arrows indicate a *tgs-1 + pou2/3+* cell. (D-G) Mfuzz clustering of gene expression dynamics in the lineages of epidermal (D), intestinal (E), muscular (F), and pharyngeal (G) progenitors.

**Additional file 4: Supplemental Fig. 4**. *ski-3 + tgs-1+* cells are enriched in the putative pluripotent stem cell clusters (CS0 and CS1). (A-C) tSNE plots showing *ski-3*+ cells in blue. (D) tSNE plots showing *ski-3* + *tgs-1+* cells in blue. (E) FISH of *ski-3* with *piwi-1* in planarians. *Piwi-1*+ (magenta); *ski-3* (green); nuclei (blue); indicate channels. Scale bars indicate 100 μm. (F) FISH of *ski-3* with *tgs-1* in planarians. *tgs-1* (magenta); *ski-3* (green); nuclei (blue) indicate channels. Scale bars indicate 100 μm.

**Additional file 5: Table S1.**

**Additional file 6 Table S2.**

**Additional file 7: Table S3.**

**Additional file 8: Table S4.**

## Data Availability

The scRNA-seq datasets of SirNeoblasts are available at GEO (GSE 158706). Reagents and other datasets are available from the corresponding author on reasonable request.
