# Supplementary figures and images for: Canonical and early lineage-specific stem cell types identified in planarian SirNeoblasts
Source: Cell Regen. 2021 Mar 19;10:15. doi: 10.1186/s13619-021-00076-6 (PMC7979843; doi:10.1186/s13619-021-00076-6)

**Figure 1\_S1**

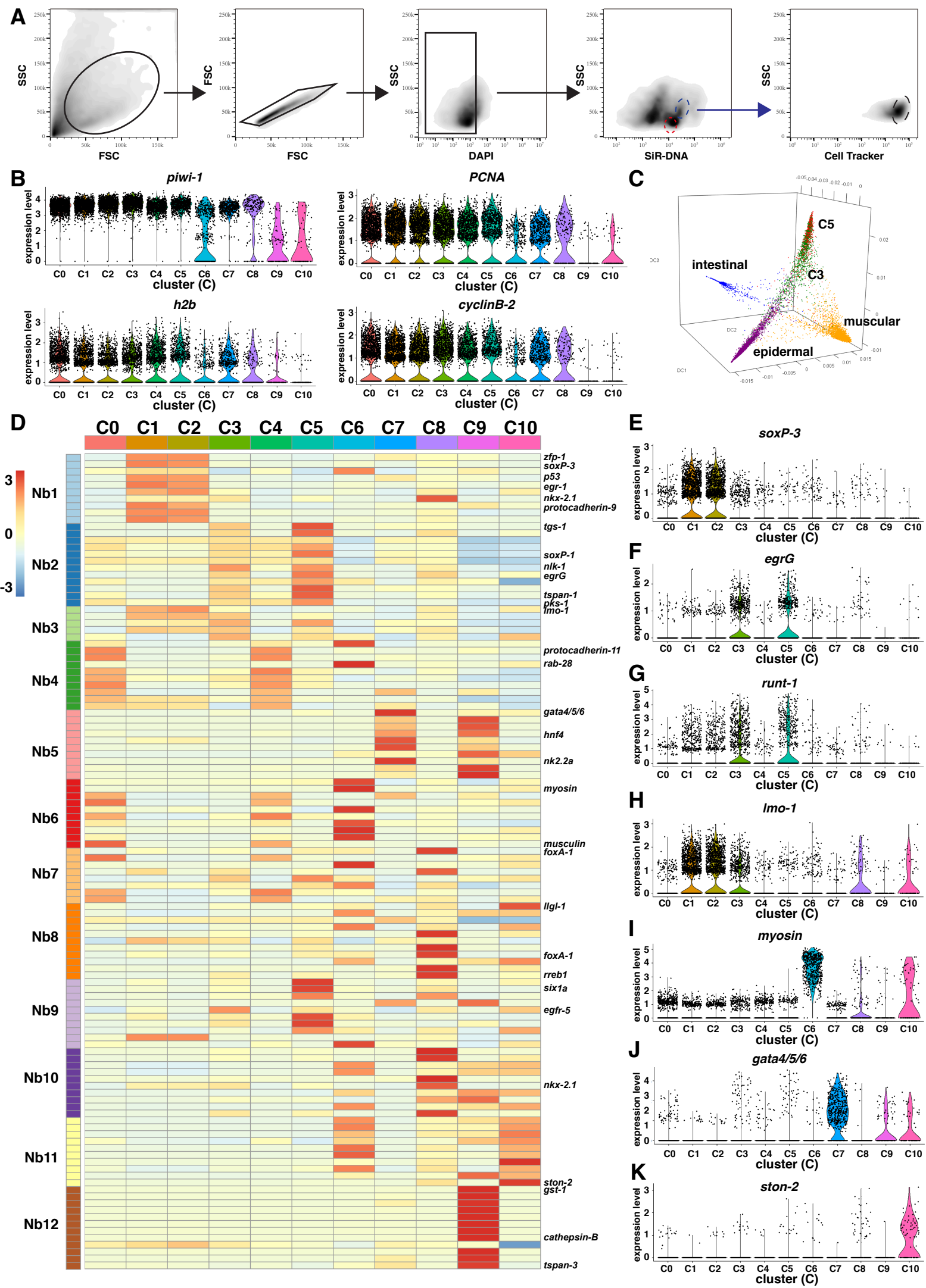

Supplement: Supplementary file 1 — Additional file 1: Supplemental Fig. 1. (A) Flow cytometry plots showing the gating steps to enrich SirNeoblasts. (B) Violin plots showing the enrichment and expression levels of pan-neoblast markers. (C) Pseudotime snapshot of lineage analysis for perspective pluripotent stem cells with epidermal (purple), intestinal (blue), muscular (orange) progenitors and C5 (red), C3 (green) populations. (D) Heatmap correlation analysis for markers from X1 scRNA-seq data. (E-K) Violin plots showing the enrichment and expression levels of each neoblast marker. [file 13619_2021_76_MOESM1_ESM.pdf]

Figure 2\_S1

A

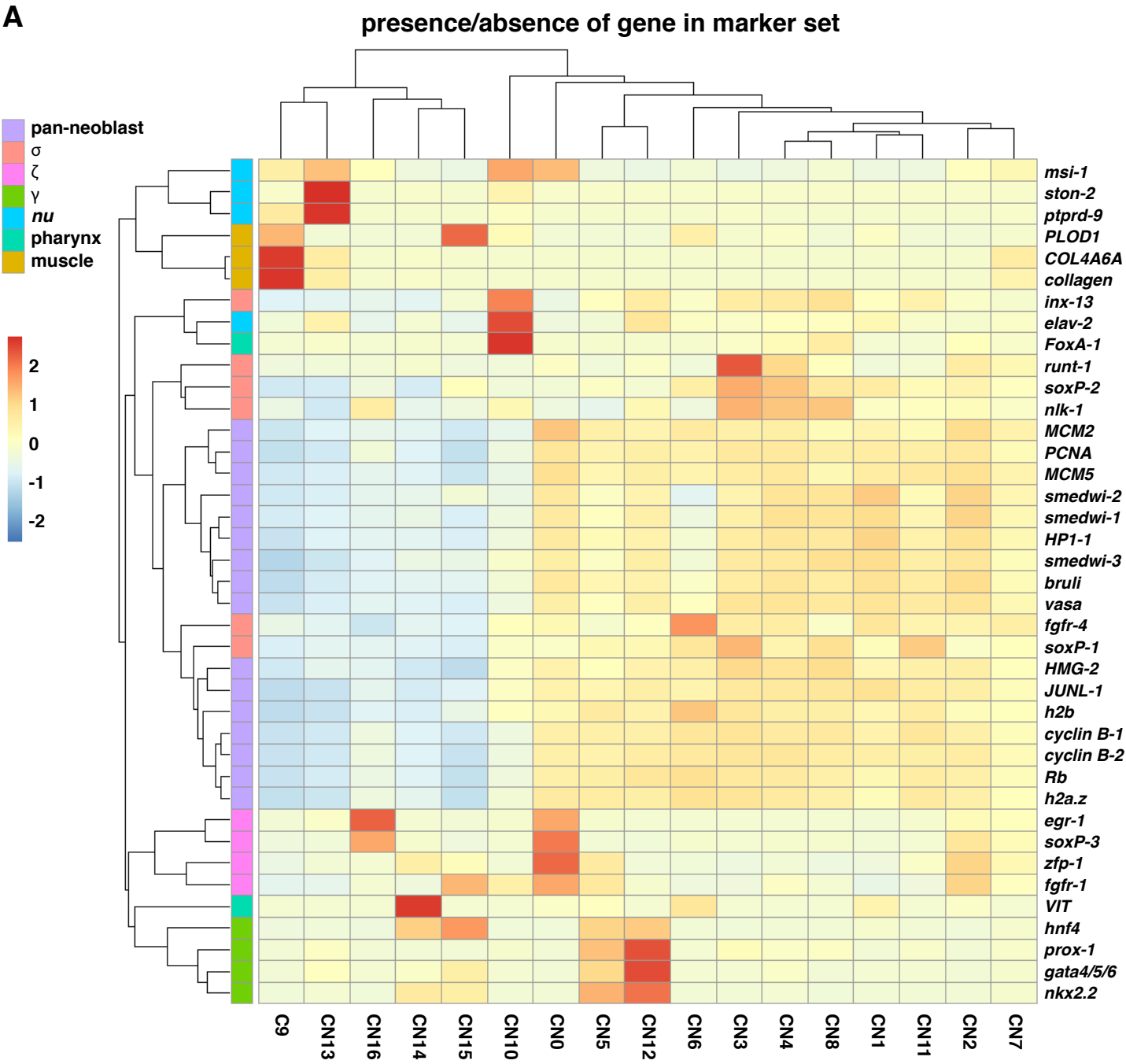

B

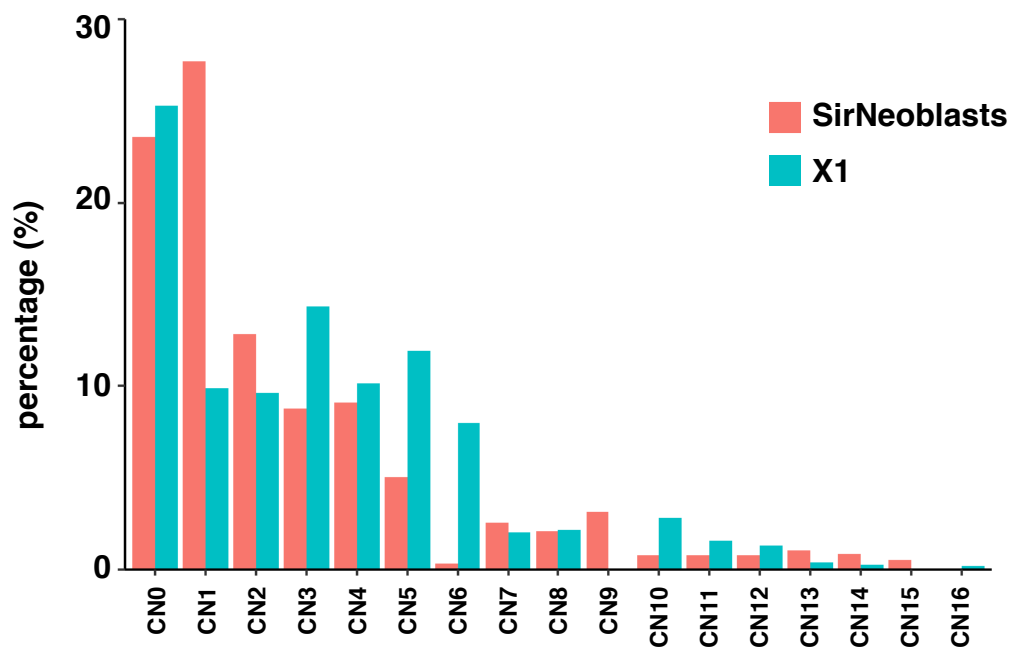

Supplement: Supplementary file 2 — Additional file 2: Supplemental Fig. 2. Verification of markers from X1 single cell data. (A) Heatmap correlation analysis for traditional markers. (B) Proportion of total cells and piwi-1+ cells distributed in each cluster. [file 13619_2021_76_MOESM2_ESM.pdf]

Figure 3\_S1

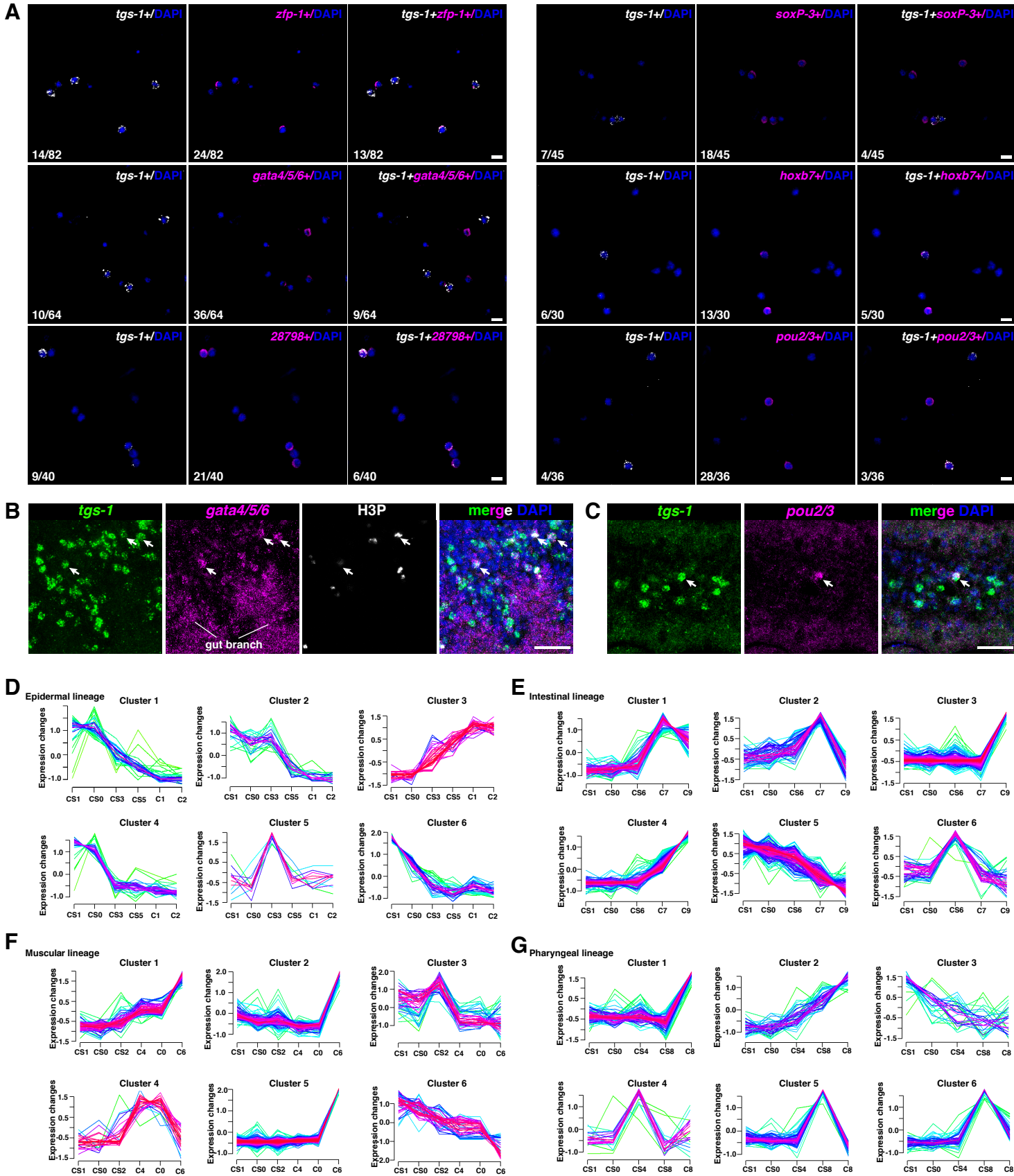

Supplement: Supplementary file 3 — Additional file 3: Supplemental Fig. 3. Gene expression dynamics in each progenitor lineage. (A) FISH of tgs-1 (white) with lineage marker genes (magenta) including zfp-1, soxP-3, gata4/5/6, hoxb7, SMED30028798 (28798), and pou2/3 in SirNeoblasts, respectively. The numbers indicate the number of positive cells versus the number of total cells counted. Scale bars indicate 10 μm. (B) FISH of gata4/5/6 (magenta) with tgs-1 (green) in planarians. Scale bars indicate 50 μm. Arrows indicate the tgs-1 + gata4/5/6+ cells. (C) FISH of pou2/3 (magenta) with tgs-1 (green) in planarians. Scale bars indicate 50 μm. Arrows indicate a tgs-1 + pou2/3+ cell. (D-G) Mfuzz clustering of gene expression dynamics in the lineages of epidermal (D), intestinal (E), muscular (F), and pharyngeal (G) progenitors. [file 13619_2021_76_MOESM3_ESM.pdf]

Figure 4\_S1

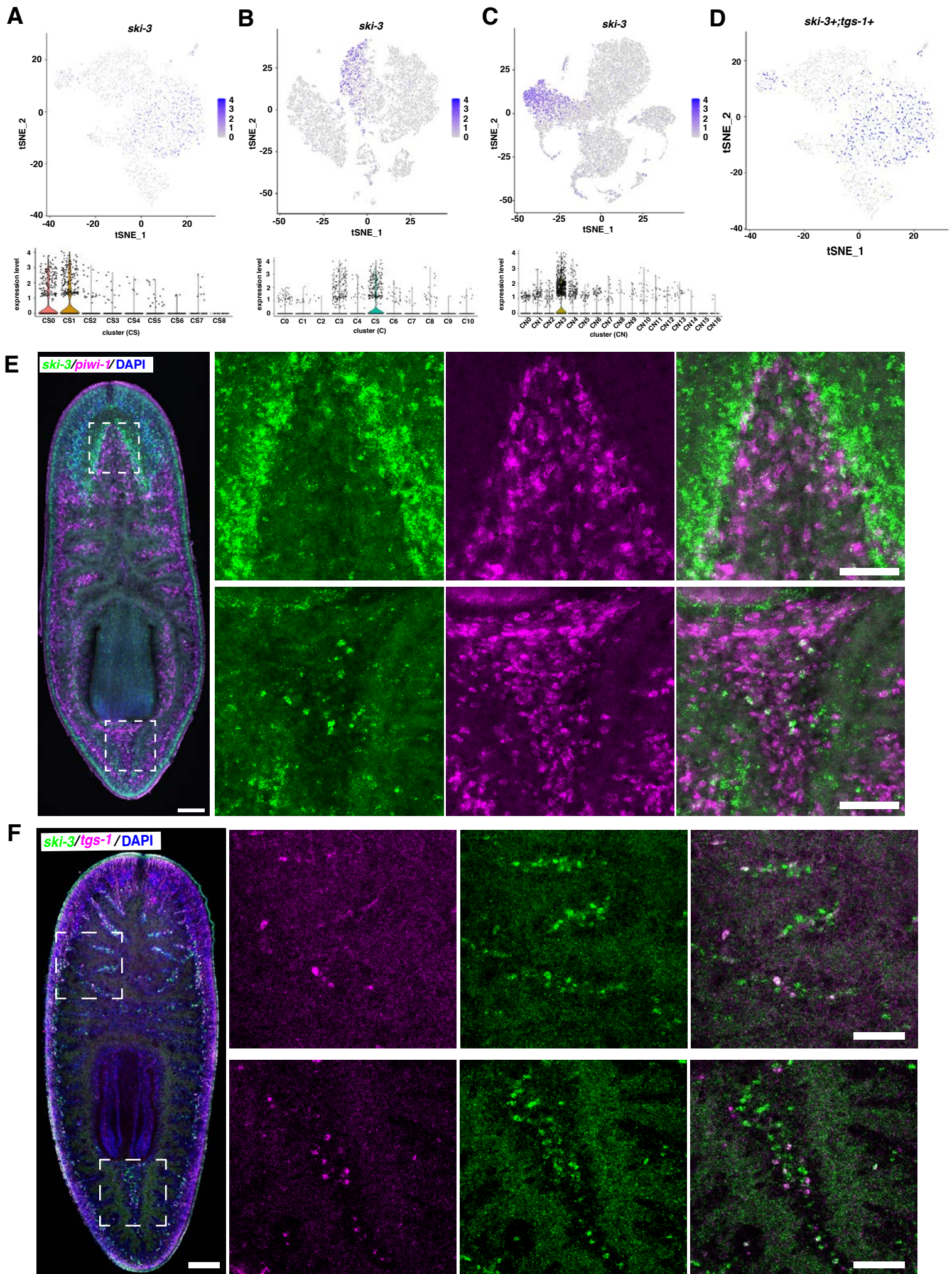

Supplement: Supplementary file 4 — Additional file 4: Supplemental Fig. 4. ski-3 + tgs-1+ cells are enriched in the putative pluripotent stem cell clusters (CS0 and CS1). (A-C) tSNE plots showing ski-3+ cells in blue. (D) tSNE plots showing ski-3 + tgs-1+ cells in blue. (E) FISH of ski-3 with piwi-1 in planarians. Piwi-1+ (magenta); ski-3 (green); nuclei (blue); indicate channels. Scale bars indicate 100 μm. (F) FISH of ski-3 with tgs-1 in planarians. tgs-1 (magenta); ski-3 (green); nuclei (blue) indicate channels. Scale bars indicate 100 μm. [file 13619_2021_76_MOESM4_ESM.pdf]
